# Impact of Wilms’ tumor 1 gene (WT1) mutation on outcome of allogeneic hematopoietic-cell transplantation for acute myeloid leukemia: a retrospective multicenter cohort study from the ALWP/EBMT registry

**DOI:** 10.1038/s41409-025-02727-7

**Published:** 2025-10-16

**Authors:** Arnon Nagler, Jordi Esteve, Jacques-Emmanuel Galimard, Jaime Sanz, Xavier Poire, Matthew Collin, Johan Maertens, He Huang, Maija Itäla-Remes, Khalid Halahleh, Maria Jesús Pascual Cascon, Patrizia Chiusolo, Laimonas Griskevicius, Ain Kaare, Ann De Becker, Pavel Jindra, Jose Antonio Pérez-Simón, Ali Bazarbachi, Eolia Brissot, Fabio Ciceri, Mohamad Mohty

**Affiliations:** 1 Division of Hematology, Sheba Medical Center, Tel Hashomer, Israel; 2 Hematology Department, Institute of Cancer & Blood Disorders (ICAMS), Hospital Clinic, IDIBAPS, University of Barcelona, Barcelona, Spain; 3 EBMT Paris Study Unit; Department of Haematology, Saint Antoine Hospital; INSERM UMR 938, Sorbonne University, Paris, France; 4 Hematology Department, Hospital Universitari i Politècnic La Fe, Valencia Departament de Medicina Universitat de Valencia, CIBERONC, Instituto Carlos III, Madrid, Spain; 5 Cliniques Universitaires St. Luc, Brussels, Belgium; 6 Royal Victoria Infirmary, Newcastle, UK; 7 University Hospital Gasthuisberg, Leuven, Belgium; 8 First Affiliated Hospital of Zhejiang University School of Medicine, Hangzhou, China; 9 Turku University Hospital, Turku, Finland; 10 King Hussein Cancer Centre Adult BMT Program, Amman, Jordan; 11 Hospital Regional de Málaga, Malaga, Spain; 12 Ematologia e Trapianto-Dipartimento di Scienze di Laboratorio ed Ematologiche-Fondazione Policlinico Universitario Agostino Gemelli IRCCS-Sezione di Ematologia-Università Cattolica del Sacro Cuore, Roma, Italy; 13 Vilnius University Hospital Santaros Klinikos, Vilnius, Lithuania; 14 Tartu University Hospital, Tartu, Estonia; 15 Universiteit Brussel (VUB), Universitair Ziekenhuis Brussel (UZ Brussel), Hematology Department, Brussels, Belgium; 16 Charles University Hospital, Pilsen, Czechia; 17 Hospital Universitario Virgen del Rocío, Seville, Spain; 18 Hematology-Oncology Division, Department of Internal Medicine, American University of Beirut Medical Center, Beirut, Lebanon; 19 Service d’Hématologie clinique et Thérapie cellulaire, Hôpital Saint-Antoine, APHP, Paris, France; 20 Ospedale San Raffaele, Haematology and BMT, Milano, Italy; 21 Sorbonne University, Department of Haematology, Saint Antoine Hospital, Paris, France

**Keywords:** Acute myeloid leukaemia, Oncogenesis

## Abstract

We compared transplantation outcomes of AML patients with *WT1* mutation (mWT1), identified by next-generation sequencing, to those of patients with wild-type *WT1* AML (wtWT1). 703 patients were included, 50 with mWT1 and 653 with wtWT1. Patients with mWT1 were younger (median age: 45.6 vs. 56.4 years, *p* < 0.001), with a higher proportion of females (66% vs. 47.6%, *p* = 0.01), higher frequency of mutations in *FLT3-ITD* (38.3% vs. 21.7%, *p* = 0.01) and *CEBPA* (15.8% vs. 5.7%, *p* = 0.03). Donors were matched siblings in 30.6%, unrelated in 45.6%, and haploidentical in 22.1%. A higher percentage of mWT1 vs. wtWT1 patients received in vivo T-cell depletion (66% vs. 51%, *p* = 0.03) and 58% vs. 47.1% received myeloablative conditioning. 49 patients with mWT1 were matched to 127 wtWT1 patients in matched-pairs analysis. Outcomes (mWT1 vs. wtWT1) were not significantly different: relapse (2 y: 28.8% vs. 30.4%, HR: 1.14, *p* = 0.64), NRM (2 y: 15.5% vs. 9.9%, HR: 1.41, *p* = 0.49), LFS (2 y: 55.7% vs. 59.6%, HR: 1.21, *p* = 0.39), OS (2 y: 65.4% vs. 73.3%, *p* = 0.66), and chronic GVHD (2 y:24.3% vs. 25.4%, *p* = 0.95). In conclusion, *WT1* mutation did not influence transplantation outcomes of AML patients in CR1.

## Introduction

Prognosis of patients with acute myeloid leukemia (AML), treated with any of currently available options (i.e., either intensive chemotherapy or low intensity regimens), the success rate of the induction and post-induction therapies and, as a consequence, indication of allogeneic stem cell transplantation (HSCT), are mainly dictated by leukemic-associated biological features, especially cytogenetics and mutation profile, recently defined by the next generation sequencing (NGS) technique [1–9]. The Wilms tumor 1 (*WT1*) gene is a transcription factor, located on chromosome 11p13, which encodes for a zinc finger protein that has been involved in the regulation of cell survival, proliferation, and differentiation, and may function both as a tumor suppressor and an oncogene [6, 7]. Approximately 5–10% of AML patients carry *WT1* gene mutations, affecting mainly hotspots in exons 7 and 9, and less frequently in other exons such as 1, 2, 3, and 8 [10–13]. Mutations in the *WT1* gene cause conformational changes in the binding capacity of the WT1 protein, leading to a deficient tumor-suppressing activity and creating a pro-tumor environment [13]. The *WT1* gene, in addition, is overexpressed in 75–100% of adult AML patients, and its high expression is associated with different molecular alterations such as FMS-like tyrosine kinase-3 internal tandem duplication (FLT3-ITD), nucleophosmin 1 *(NPM1)* mutation, *CBFb: MYH11* gene fusion, as well as *KMT2A* rearrangement, while a low expression is observed in *RUNX1:RUNX1T1* AML. Consequently, *WT1* overexpression has been widely used as a measurable residual disease (MRD) marker [14–19]. *WT1* mutation has been identified as an independent predictor of worse clinical outcomes in adult AML in some studies [14–16], while other studies suggest a lack of significant impact [17–19]. Moreover, recently, Tazi Y et al. proposed a distinctive genetic category of AML mainly defined by the presence of *WT1* mutations in the absence of other genetic subtype-defining events associated with poor outcome, comparable to that of other adverse-risk features, when associated with *FLT3*-ITD co-mutation [7]. The *WT1* mutation appears to be more prevalent among patients diagnosed with AML with *CEBPA*-bZIP or double *CEBPA* mutation, and the concurrence of *WT1* mutation could worsen the prognosis of some favorable-risk subsets, such as AML with *NPM1* and AML associated with *CEBPA*-bZIP mutation [19].

However, data regarding the impact of mWT1 on HSCT outcome are rather limited. Several studies have observed an independent higher cumulative risk of relapse after the procedure, including a very recent study based on 56 patients with mWT1 AML from a large series of 6887 patients with myeloid malignancies, 25 of whom underwent HSCT [20]. Similarly, Pan X [19] reported a higher relapse incidence (RI) specifically conferred by *WT1* mutation in the subset of European LeukemiaNet (ELN) intermediate-risk patients. A higher RI has also been reported in a few additional studies [21, 22]. Finally, Atluri H recently suggested a potential beneficial effect of HSCT in intermediate-risk patients harboring a *WT1* mutation [23].

Given this uncertain impact of *WT1* mutation on transplant outcome, we performed a contemporary registry-based real-life study using data of the Acute Leukemia Working Party (ALWP) of the European Society for Blood and Marrow Transplantation (EBMT), comparing the outcomes of HSCT in patients with mWT1 AML in first complete remission (CR1) with that of patients with wild-type *WT1* (wtWT1).

## Patients and methods

### Study design and data collection

This was a retrospective, multicenter analysis using the dataset of the ALWP of the EBMT. The EBMT is a voluntary working group of more than 600 transplant centers that are required to report all consecutive stem cell transplantations and follow-ups once a year. EBMT minimum essential data forms are submitted to the registry by transplant center personnel following written informed consent from patients per the centers’ ethical research guidelines. The study was approved by the ALWP of the EBMT, the Chaim Sheba Medical Center Helsinki committee (SMC-770-920), and the Israeli Ministry of Health. It was performed in compliance with the Declaration of Helsinki and under the guidance of the EBMT. All patients provided written informed consent authorizing the use of information for research purposes. Accuracy of data is assured by individual transplant centers and by quality control measures such as regular internal and external audits. The results of disease assessments at HSCT were also submitted and form the basis of this report. The study was conducted in accordance with the STROBE recommendations. All methods were performed in accordance with the relevant guidelines and regulations. Eligibility criteria for this analysis included adult patients ≥18 years of age with AML in CR1 with information on the *WT1* mutation assessed by NGS at the time of diagnosis (within 30 days from diagnosis) and who underwent a first HSCT without previous autologous HSCT between 2015 and 2023. All donor types and sources of cells were included. Data collected included recipient and donor characteristics (age, gender, and cytomegalovirus [CMV] serostatus), recipient Karnofsky performance status (KPS), disease characteristics including cytogenetic risk classification as per ELN 2022, additional mutations defined by NGS, type of AML (secondary versus de novo), pre-HSCT measurable residual disease (MRD) status, year of transplant, type of conditioning regimen, stem cell source, and graft-versus-host disease (GVHD) prophylaxis regimen. The conditioning regimen was defined as myeloablative (MAC) or reduced-intensity (RIC) based on the reports from individual transplant centers as per previously established criteria [24]. The conditioning regimen was defined as MAC when containing total body irradiation (TBI) with a dose >6 Gray or a total dose of busulfan (Bu) > 8 mg/kg or >6.4 mg/kg when administered orally or intravenously, respectively, or a total dose of treosulfan ≥36 gr/m^2^. All other regimens were defined as RIC [24]. Regimens for GVHD prophylaxis were per institutional protocols. Grading of acute (a) and chronic (c) GVHD was performed using established criteria [25, 26]. For this study, all necessary data were collected according to the EBMT guidelines, using the EBMT minimum essential data forms. The list of institutions contributing data to this study is provided in Supplementary Appendix.

### Statistical analysis

Median values and interquartile ranges (IQR) were used for quantitative variables, and frequencies and percentages were used for categorical variables. The study endpoints were overall survival (OS), leukemia-free survival (LFS), relapse incidence (RI), NRM, neutrophil recovery, aGVHD, and cGVHD. All endpoints were measured from the time of transplantation. Neutrophil recovery was defined as achieving an absolute neutrophil count (ANC) of ≥0.5 × 10^9^/L for three consecutive days. OS was defined as the time to death from any cause. LFS was calculated from the day of HSCT until disease recurrence or disease progression, death from any cause, or last follow-up. NRM was defined as death from any cause without previous relapse or progression. Patient-, disease-, and transplant-related characteristics were compared between the groups according to the presence or absence of the WT1 mutation, using the Mann–Whitney *U* test for quantitative variables, and the chi-squared or Fisher’s exact test for categorical variables. The probabilities of OS and LFS were calculated using the Kaplan–Meier estimator [27]. Neutrophil recovery, aGVHD, cGVHD, RI, and NRM were calculated using cumulative incidence curves in a competing risk setting, with death in the absence of relapse being treated as a competing event for relapse. Death was considered a competing event for engraftment. To estimate the cumulative incidence of aGVHD or cGVHD, relapse, and death were considered as competing events.

In order to have comparable groups on which to estimate the impact of WT1 on outcomes, a matched-pairs analysis was performed [28]. Donor type and source of cells were used for exact matching, and matching on year of HSCT, patient age, and sex at HSCT, as well as in vivo T-cell depletion, were based on a propensity score model [28]. Of the 50 patients with mWT1, 36 were matched with 3 wtWT1 patients, 6 were matched with 2 pairs of patients, 7 with 1 pair, and 1 mWT1 patient was not matched.

The impact of *WT1* post pair-matching was estimated using the Cox proportional-hazards regression model, including a cluster on the pairs in the models [27–29]. Results are expressed as the hazard ratio (HR) with a 95% confidence interval (95% CI). Due to the low number of patients with mutated WT1, no variables with missing data on these patients were included in the propensity score matching, and no subgroup analysis was performed.

All *p*-values were two-sided with a type 1 error rate fixed at 0.05. Statistical analyses were performed with R 4.2.3 (R Core Team Fifty 2020). R: A language and environment for statistical computing. R Foundation for Statistical Computing, Vienna, Austria. URL 8832 https://www.R-project.org/ [30].

## Results

### Patient, disease, and transplant characteristics

Seven hundred and three patients met the study inclusion criteria, 50 with mWT1 and 653 with wtWT1 (Table 1). Median follow-up was 3 years (95% CI: 2.9–3.1). The median year of transplantation was 2020 (range, 2016–2022) and 2019 (range, 2015–2023), respectively (*p* = 0.84). Patients with mWT1 were younger (median age: 45.6 vs. 56.4 years, *p* < 0.001), and a higher proportion were female (66% vs. 47.6%, *p* = 0.01). Approximately 77% of patients in total presented with de novo AML, without a significant difference between the two groups. Among mWT1 patients, a higher frequency of mutations in *CEBPA* (15.8% vs. 5.7%, *p* = 0.03) and *FLT3*-ITD (38.3% vs. 21.7%, *p* = 0.01) genes was observed (Supplemental Table S1), as well as a non-significant lower proportion of ELN 2022-defined adverse risk category cytogenetics (17.9 vs. 28.6, *p* = 0.11) (Table 1). Time from diagnosis to HSCT was 4.7 months in each group. In the total cohort, donors were matched siblings in 30.6%, unrelated in 44.6%, and haploidentical in 22.1%. A higher (but non-significant) percentage of mWT1 compared to wWT1 patients received MAC (58% vs. 47.1%, *p* = 0.14) (Table 1), with Bu/fludarabine being the most frequent regimen for the mWT1 group (36%) and Bu/fludarabine/thiotepa for the wWT1 group (29.4%) (Supplemental Table S2). In vivo T-cell depletion was used in a higher proportion of mWT1 patients (66% vs. 51.2%, respectively, *p* = 0.03). Regarding GVHD prophylaxis, a cyclosporine A/mycophenolate mofetil-based regimen was used in 32% vs. 26.6%, mycophenolate mofetil/sirolimus/tacrolimus in 32% vs. 30.8%, and post-transplant cyclophosphamide in 37% vs. 38% of mWT1 and wtWT1 patients, respectively (Table 1, Supplementary Table S3). Other characteristics did not differ significantly between the groups, including KPS, CMV seropositivity, female donor to male patient combination, and stem cell source (peripheral blood was used in 96% vs. 93.3% of patients). Patient and transplant characteristics, additional mutations, detailed conditioning regimens, and GVHD prevention by matched-pair analysis are depicted in Supplemental Tables S4–S7, respectively.

**Table 1:** Patient and transplant characteristics.

Variables		All (*N* = 703)	wtWT1 (*n* = 653)	mWT1 (*n *= 50)	*p* value
Year of HSCT	median [IQR]	2019 [2018–2021]	2019 [2018–2021]	2020 [2018–2021]	0.84
	(range)	(2015–2023)	(2015–2023)	(2016–2022)	
Age at HSCT (years)	median [IQR]	55.7 [43.2–63]	56.4 [43.8–63]	45.6 [35.7–56]	<0.001
	(range)	(18.2–75.1)	(18.2–75.1)	(20.3–70.2)	
Patient sex *n* (%)	Female	344 (48.9)	311 (47.6)	33 (66)	0.01
	Male	359 (51.1)	342 (52.4)	17 (34)	
Interval diagnosis-HSCT (months)	median [IQR]	4.7 [4–6]	4.7 [4–6]	4.7 [3.7–6.2]	0.79
	(range)	(0.9–23.5)	(0.9–23.5)	(1.7–23.4)	
AML type *n* (%)	de novo	539 (76.7)	498 (76.3)	41 (82)	0.47
	secAML	164 (23.3)	155 (23.7)	9 (18)	
Cytogenetics ELN-2022 *n* (%)	Favorable	29 (5)	25 (4.6)	4 (10.3)	0.11f
	Intermediate	387 (67.1)	359 (66.7)	28 (71.8)	
	Adverse	161 (27.9)	154 (28.6)	7 (17.9)	
	Missing	127	116	11	
*FLT3*-ITD *n* (%)	Present	154 (22.9)	136 (21.7)	18 (38.3)	
	Absent	519 (77.1)	490 (78.3)	29 (61.7)	
	Unknown	31	28	3	0.01
*CEBPA* mutation *n* (%)	Present	37 (6.4)	31 (5.7)	6 (15.8)	
	Absent	545 (93.6)	513 (94.3)	32 (84.2)	
	Unknown	121	109	12	0.03f
MRD *n* (%)	Positive	127 (32.3)	117 (32.9)	10 (27)	0.47
	Negative	266 (67.7)	239 (67.1)	27 (73)	
	missing	310	297	13	
KPS *n* (%)	<90	131 (19.3)	122 (19.4)	9 (18.4)	0.86
	>= 90	547 (80.7)	507 (80.6)	40 (81.6)	
	missing	26	25	1	
Myeloablative regimen *n* (%)	No	358 (52.9)	337 (52.9)	21 (42)	0.14
	Yes	329 (47.9)	300 (47.1)	29 (58)	
	missing	16	16	0	
In-vivo TCD *n* (%)	No	332 (47.7)	315 (48.8)	17 (34)	0.03 (No
	ATG	357 (51.3)	324 (50.2)	33 (66)	In vivo vs
	Alemtuzumab	7 (1)	7 (1.1)	0 (0)	In vivo)
	Missing		7	0	
PTCy *n* (%)	No	435 (63)	404 (63)	31 (62)	0.88
	Yes	256 (37)	237 (37)	19 (38)	
	Missing	12	12	0	
Source of cells *n* (%)	PB	657 (93.5)	609 (93.3)	48 (96)	
	BM	38 (4.4)	36 (5.5)	2 (4)	1f
	Other	8 (1.1)	8 (1.2)	0 (0)	
Donor type *n* (%)	Matched sibling	215 (30.6)	201 (30.8)	14 (28)	Not done
	Unrelated	320 (44.6)	293 (44.9)	27 (54)	
	*HLA 10/10 match*	*233 (33.2)*	*214 (32.8)*	*19 (38)*	
	*HLA 9/10 match*	*42 (6)*	*34 (5.2)*	*8 (16)*	
	*HLA* ≤ *8/10 match*	*2 (0.3)*	*2 (0.3)*	*0*	
	*Unknown HLA match*	*36 (5.1)*	*36 (5.5)*	*0*	
	Unrelated CB	*7 (1)*	*7 (1.1)*	*0*	
	Haploidentical	155 (22.1)	148 (22.7)	7 (14)	
	Other	12 (1.7)	10 (1.5)	2 (4)	
	Missing	1	1	0	
Female to Male *n* (%)	No	592 (84.1)	547 (83.6)	45 (90)	0.24
	Yes	112 (15.9)	107 (16.4)	5 (10)	
Patient CMV *n* (%)	Negative	180 (26.3)	163 (25.7)	20 (40)	0.2
	Positive	504 (73.7)	471 (74.3)	33 (66)	
	missing	19	19	0	
Donor CMV *n* (%)	Negative	291 (42.5)	271 (42.7)	20 (40)	0.71
	Positive	393 (57.5)	363 (57.3)	30 (60)	
	missing	19	19	0	

*wtWT1* wild type Wilms tumor 1, *mWT1* mutated Wilms tumor 1, *IQR* interquartile range, *HSCT* hematopoietic stem cell transplantation, *AML* acute myeloid leukemia, *secAML* secondary acute myeloid leukemia, *HLA* human leukocyte antigen, *CMV* cytomegalovirus, *BM* bone marrow, *PB* peripheral blood, *CB* cord blood, *KPS* Karnofsky performance score, *TCD* T-cell depletion, *ATG* anti-thymocyte globulin, *ELN* European Leukemia Net, *FLT3* *ITD* *FMS* like tyrosine kinase 3 internal tandem duplication, *CEBPA* *CCAAT* enhancer binding protein A gene, *PTCy* post transplantation cyclophosphamide, *MRD* measurable residual disease, *f* fisher exact test.

### Transplantation outcomes

**Fig. 1:**
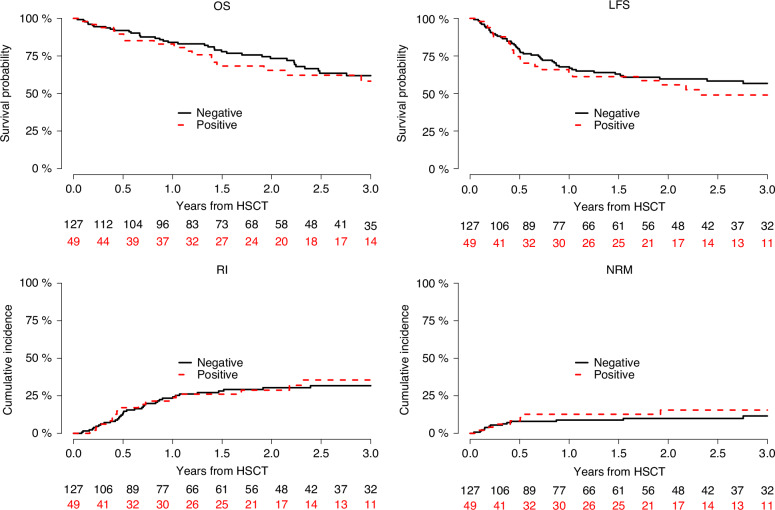
Matched-pair analysis of hematopoietic stem cell transplant (HSCT) outcomes in acute myeloid leukemia (AML) patients with WT1 mutation (mWT1) versus AML patients without WT1 mutation (wtWT1). OS overall survival, LFS leukemia-free survival, RI relapse incidence, NRM nonrelapse mortality.

Day 30 ANC recovery was 98.4% versus 96.7% in mWT1 and wtWT1, respectively (Table 2). Incidence of both acute and cGVHD were not significantly different between the 2 cohorts: day 100 aGVHD Grade II-IV 16.7% (95% CI 7.7–28.5) versus 25.1% (95% CI 17.7–33.1), HR  =  0.74 (95% CI 0.34–1.61, *p*  =  0.45), aGVHD Grade III-IV 2.1% (95% CI 0.2–9.7) versus 9.2% (95% CI 4.9–15.2), HR  =  0.22 (95% CI 0.03–1.83, *p*  =  0.16), and 2 years cGVHD 24.3% (95% CI 12.9–37.7) versus 25.4% (95% CI 17.6–33.9), HR  =  1.02 (95% CI 0.5–2.11, *p*  =  0.95), respectively (Tables 2 and 3). There were no significant differences between the groups with respect to two-year NRM and RI: 15.5% (95% CI 6.6–27.8) versus 9.9% (95% CI 5.4–16.1%), HR  =  1.41 (95% CI 0.54–3.67, *p*  =  0.49), and 28.8% (95% CI 16.3–42.5%) versus 30.4% (95% CI 22.1–39.1%), HR  =  1.14 (95% CI 0.66–1.97, *p*  =  0.64), respectively (Tables 2 and 3, Fig. 1). There were no significant differences in LFS and OS between the mWT1 versus wtWT1 groups: 55.7% (95% CI 39.9–69%) versus 59.6% (95% CI 50–68%), HR  =  1.21 (95% CI 0.78–1.87, *p*  =  0.39) and 65.4% (95% CI 49–77.6%) versus 73.3% (95% CI 63.7–80.7%), HR  =  1.14 (95% CI 0.64–2.09, *p*  =  0.66), respectively (Table 3, Fig. 1).

**Table 2:** Outcomes post pair-matching.

Outcomes	All	wtWT1	mWT1
	Estimation (95% CI)	Estimation (95% CI)	Estimation (95% CI)
Median FU (y)	2.8 (2.3–3.3)	2.7 (2.1–3.3)	2.9 (2.1–3.5)
OS (2 y)	71 (62.9–77.6)	73.3 (63.7–80.7)	65.4 (49–77.6)
LFS (2 y)	58.6 (50.5–65.9)	59.6 (50–68)	55.7 (39.9–69)
RI (2 y)	29.9 (22.9–37.2)	30.4 (22.1–39.1)	28.8 (16.3–42.5)
NRM (2 y)	11.5 (7.2–17)	9.9 (5.4–16.1)	15.5 (6.6–27.8)
Poly recovery (30 d)	97.1 (92.8–98.8)	96.7 (90.9–98.8)	98.4 (72–99.9)
aGVHD-II/IV (100 d)	22.6 (16.6–29.2)	25.1 (17.7–33.1)	16.7 (7.7–28.5)
aGVHD-III/IV (100 d)	7.2 (3.9–11.7)	9.2 (4.9–15.2)	2.1 (0.2–9.7)
cGVHD (2 y)	25.1 (18.6–32.2)	25.4 (17.6–33.9)	24.3 (12.9–37.7)

*wtWT1* wild type Wilms tumor 1, *mWT1* mutated Wilms tumor 1, *FU* follow up, *y* year, *d* day, *RI* relapse incidence, *NRM* non-relapse mortality, *LFS* leukemia-free survival, *OS* overall survival, *aGVHD* acute graft-versus-host disease, *cGVHD* chronic graft-versus-host disease, *Ext* extensive, *Poly* polymorphonuclear, *CI* confidence interval.

**Table 3:** Outcomes post pair-matching (Cox model, reference: wtWT1).

Outcomes	HR (95% CI)	*p* value
OS	1.14 (0.64–2.05)	0.66
LFS	1.21 (0.78–1.87)	0.39
RI	1.14 (0.66–1.97)	0.64
NRM	1.41 (0.54–3.67)	0.49
aGVHD II-IV	0.74 (0.34–1.61)	0.45
aGVHD III-IV	0.22 (0.03–1.83)	0.16
cGVHD	1.02 (0.5–2.11)	0.95

*HR* hazard ratio, *wtWT1* wild type Wilms tumor 1, *RI* relapse incidence, *NRM* non-relapse mortality, *LFS* leukemia-free survival, *OS* overall survival, *aGVHD* acute graft-versus-host disease, *cGVHD* chronic graft-versus-host disease, *CI* confidence interval.

### Cause of death

A total of 56 patients died after transplant (Table 4). Primary disease was the main cause, constituting 58.8% and 65.8% of the deaths in the mWT1 and wtWT1 groups, respectively. The second most common cause of death was infection (29.4% and 13.2%), while death due to GVHD was reported in 5.9% and 7.9%, respectively (Table 4). Other transplant-related deaths accounted for 5.9% and 13.2%, respectively. Infrequent causes of death, such as secondary malignancies and veno-occlusive disease of the liver, did not differ between the two groups (Table 4).

**Table 4:** Cause of death post-pair-matching.

Cause of death	*N* = 56	wtWT1 (*n* = 39)	mWT1 (*n* = 17)
Relapse	35 (63.6)	25 (65.8)	10 (58.8)
GVHD	4 (7.3)	3 (7.9)	1 (5.9)
Infection	10 (18.2)	5 (13.2)	5 (29.4)
Other HSCT-related	4 (7.3)	3 (7.9)	1 (5.9)
Secondary malignancy	1 (1.8)	1 (2.6)	0 (0)
VOD	1 (1.8)	1 (2.6)	0 (0)
Missing	1	1	0

*wtWT1* wild type Wilms tumor 1, *mWT1* mutated Wilms tumor 1, *HSCT* hematopoietic stem cell transplantation, *GVHD* graft-versus-host disease, *VOD* veno-occlusive disease of the liver.

## Discussion

In the current study, focusing on AML patients with *WT1* mutation undergoing transplantation from MSD, UD, or haploidentical donors, while in CR1, we assessed the impact of mWT1 on HSCT outcomes in comparison to transplantation outcomes of AML patients with wtWT1. HSCT outcome parameters, including NRM, RI, LFS, and OS, did not differ between patients with mWT*1* and those with wt*WT1*. Lack of deleterious prognostic value was further confirmed with a matched-pairs analysis after adjustment for main variables. These results, obtained in a patient population who had recently received HSCT, are important since the impact of *WT1* mutations in AML has not been completely determined and was found to be negative in previous studies.

Most, but not all, of the previously published literature has indicated a higher relapse rate and inferior survival in AML patients harboring the *WT1* mutation [14, 15, 31, 32]. Hou et al. assessed the impact of the *WT1* mutation in 470 de novo AML patients, showing that the *WT1* mutation was an independent poor prognostic factor in multivariate analysis for both OS and LFS. The authors also developed a useful survival scoring system incorporating the *WT1* mutation, *NPM1*/*FLT3*-ITD, *CEBPA* mutations, and age into survival analysis, concluding that *WT1* mutations were correlated with poor prognosis [16]. Nonetheless, the transplant rate in this study was low (20%), as well as the number of patients harboring a *WT1* mutation receiving intensive chemotherapy (*n* = 28). Similar results were reported by Renneville et al., on behalf of the Acute Leukemia French Association, who studied 268 AML patients and demonstrated that patients who had *WT1* mutation had a shorter 4-year OS and a higher risk of recurrence at 4 years compared to those of patients with wild-type *WT1* [14]. Nonetheless, the number of transplants in CR1 among patients with *WT1*-mutated AML was low (2 out of 14). Colleagues from the United Kingdom Medical Research Council Adult Leukemia Working Party performed a similar study, comparing the results in 470 adults treated with conventional therapy according to *WT1* mutation and demonstrating that patients with *WT1* mutations had an inferior response to induction chemotherapy, a higher rate of resistant disease, an increased rate of relapse, and an inferior 5-year relapse-free survival (RFS) and OS compared with wild-type cases [31]. In a multivariate analysis, which also included *FLT3–ITD* and *NPM1* mutation status, the presence of *WT1* mutation remained an independent adverse prognostic factor [31]. Again, the number of *WT1* patients who underwent an allogeneic HSCT was low in this study (7 out of 47). Similarly, in a Cancer and Leukemia Group B study, which included 196 adult AML patients from two clinical studies, WT*1* mutation predicted a worse 3-year disease-free and OS compared to wild-type *WT1* AML patients independently of *CEBPA*, *FLT3*-ITD, and *NPM1* mutational status [32]. In agreement, a recent meta-analysis of 7 studies assessed the prognostic significance of *WT1* mutations in adults with AML and reported that the remission rate of patients with *WT1* mutations was inferior compared to that of patients with wild-type WT1 [33]. In contrast, other studies have failed to observe a deleterious prognostic effect related to *WT1* mutation. The German-Austrian AML Study Group (AMLSG) analyzed the impact of the *WT1* mutation in 617 AML patients and failed to show any difference in RFS and OS between patients with or without *WT1* mutations. Subset analysis showed that patients with *WT1* mutation and concomitant *FLT3*-ITD had a worse outcome in terms of CR rate, RFS, and OS, compared with patients with *WT1*-mutated AML without *FLT3*-ITD co-mutation. [34]. In the US, Ho PA et al., on behalf of the Children’s Oncology Group, screened 842 AML patients with diagnostic bone marrow specimens treated in three consecutive pediatric AML trials for *WT1* mutations. Patients had similar rates of CR, OS, and LFS regardless of *WT1* mutation, unless they harbored concomitant WT1 and FLT3-ITD mutation. The authors concluded that, similarly to the previous AMLSG study among adult AML patients, the presence of *WT1* mutation did not confer an independent negative prognostic impact in pediatric AML [18]. Difference in results obtained by all these studies may be due to diversity in the patient populations, with respect to age, underlying cytogenetics and mutational landscape, treatment received, study period, and analysis of possible confounding factors, such as interaction with co-mutations with a known prognostic value, such as *FLT3*-ITD. Importantly, the rate of allogeneic HSCT in first CR was low in most of these studies, and this variable can strongly influence the relapse risk of WT1-mutated AML. Moreover, the limited size of the target population may explain this diversity in results. Finally, the wide time span encompassed by these studies is an important confounding factor, with relevant changes in general improvement observed in more recent years, due to wider access to HSCT and overall improvement of transplant outcomes. The use of FLT3 inhibitors in FLT3-mutated AML, a frequent co-mutation among the population of interest, is an important potential difference [16, 35].

As observed in previous studies, patients with mWT1 were younger, more frequently female, and harbored a higher frequency of mutations in *CEBPA* and *FLT3*-ITD genes [16, 34, 35]. In addition, they were possibly less frequently classified as adverse-risk cytogenetics according to the ELN 2022 stratification. Our analyses were adjusted for these variables (gender and age) as they may have had significant prognostic value.

Our results are of special note as they are in the setting of transplantation, where previous data are rather limited, corresponding to small cohort data published in the form of an abstract or letter to the editor [19, 21]. A recent Chinese analysis (discussed above) observed a higher RI after HSCT in the 58 patients with an ELN 2022 intermediate-risk harboring *WT1* mutation, compared to similar patients with a wild-type gene configuration [19]. In a recent study, Quek et al. reported an increased relapse risk post-transplant in patients with detectable *WT1* mutation before HSCT [3]. In another study, colleagues from the MD Anderson Cancer Center investigated the impact of *WT1* mutation in 67 patients with NPM1-mutated AML. An emergent *WT1* mutation, not present at diagnosis, was observed in 4 out of 15 patients who relapsed, three of whom had undergone HSCT [22]. In a similar context, patients diagnosed with MDS harboring *WT1* mutation (*n* = 20) showed a significantly higher incidence of relapse post-HSCT compared to patients with wild-type WT1 (*n* = 116; 39) [36]. In our current study, we did not observe higher post-HSCT RI in mWT1, nor could we detect any difference in HSCT outcome parameters.

The study has several limitations, including the small number of patients with WT1 with positive mutations. Being a retrospective and registry-based study, the study has additional several limitations, including the risk of selection bias, the lack of a non-transplant arm, and potentially relevant but unavailable data (such as frontline therapy, pre-HSCT MRD, and hematopoietic cell transplantation-specific comorbidity index), as well as missing cytogenetic and molecular data which made it impossible to include the ELN2022 cytogenetic/molecular risk in the propensity score model. We thus could not perform a focused analysis in determined subgroups to assess the prognostic role of *WT1* mutation in each ELN 2022 category and within specific genetic subgroups, such as *NPM1*-mutated or bZIP-mutated *CEBPA* AML. In addition, the study included transplants performed between 2015 to 2023, a rather long treatment era, and treatment options that might have occurred and impacted results during this period were not captured.

In conclusion, this large registry-based retrospective analysis did not show a negative impact of *WT1* mutation among patients who received HSCT in first CR.

## Supplementary information

Supplementary tables

## Data Availability

AN, JEG, and MM had full access to all the data in the study (available upon data-specific request).
