# Supplementary material for: Impact of Wilms’ tumor 1 gene (WT1) mutation on outcome of allogeneic hematopoietic-cell transplantation for acute myeloid leukemia: a retrospective multicenter cohort study from the ALWP/EBMT registry
Source: Bone Marrow Transplant. 2025 Oct 16;61(1):44–50. doi: 10.1038/s41409-025-02727-7 (PMC12819150; doi:10.1038/s41409-025-02727-7)
Supplement: Supplementary file 1 — Supplementary tables [file 41409_2025_2727_MOESM1_ESM.pdf]

Supplementary Appendix. Contributing Centers.

663	University Hospital La Fe	Valencia	Spain
234	Cliniques universitaires Saint-Luc	Brussels	Belgium
276	RVI Newcastle	Newcastle	United Kingdom
775	Hopital Saint Antoine	Paris	France
209	University Hospital Gasthuisberg	Leuven	Belgium
401	First Affiliated Hospital of Zhejiang University School of Medicine	Kunming	China
225	Turku University Hospital	Turku	Finland
580	King Hussein Cancer Centre Adult BMT Program	Amman	Jordan
576	Hospital Regional de Málaga	Malaga	Spain
307	Universita Cattolica del Sacro Cuore	Rome	Italy
644	Vilnius University Hospital Santaros Klinikos	Vilnius	Lithuania
746	Tartu University Hospital	Tartu	Estonia
630	Universitair Ziekenhuis Brussel	Brussels	Belgium
718	Charles University Hospital	Pilsen	Czech Republic
769	Hospital Universitario Virgen del Rocío	Sevilla	Spain
264	Hopital La Miletrie	Poitiers	France
369	American University of Beirut Medical Center	Beirut	Lebanon
282	Hospital Clínico de Valencia	Valencia	Spain
996	Antwerp University Hospital (UZA)	Antwerp E	Belgium
215	Institut Jules Bordet	Brussels	Belgium
309	Fundación Jiménez Díaz	Madrid	Spain
613	ICO-Hospital Universitari Germans Trias i Pujol	Badalona	Spain
602	Klinikum Bremen-Mitte	Bremen	Germany
559	Hospital Univ. Virgen de las Nieves	Granada	Spain
819	Hospital General Universitario Gregorio Marañón	Madrid	Spain
331	European Institute of Oncology	Milano	Italy
537	Hospital Universitario de Gran Canaria Dr. Negrin	Las Palmas	Spain
642	Hospital Universitario Central de Asturias	Oviedo	Spain
650	CHRU	Angers	France
672	ICANS - Institut de cancérologie Strasbourg Europe	Strasbourg	France
726	University of Liege	Liege	Belgium
272	Hopital Bretonneau	Tours	France
652	Hospital Cardinale Giovanni Panico	Tricase	Italy

Supplemental Table S1: Additional mutations (before pair-matching)

Variables	Modalities	N=703	wtWT1 Neg (n=653)	mWT1 Pos (n=50)	p-value
CEBPA	Negative	545 (93.6)	513 (94.3)	32 (84.2)	0.03 f
	Positive	37 (6.4)	31 (5.7)	6 (15.8)	
	missing	121	109	12	
FLT3ITD	Negative	519 (77.1)	490 (78.3)	29 (61.7)	0.01
	Positive	154 (22.9)	136 (21.7)	18 (38.3)	
	missing	31	28	3	
NPM1	Negative	546 (81.7)	509 (82)	37 (78.7)	0.58
	Positive	122 (18.3)	112 (18)	10 (21.3)	
	missing	35	32	3	
ASXL1	Negative	632 (90)	583 (89.4)	49 (98)	0.049 f
	Positive	70 (10)	69 (10.6)	1 (2)	
	missing	1	1	0	
RUNX1	Negative	584 (83.1)	543 (83.2)	41 (82)	0.83
	Positive	119 (16.9)	110 (16.8)	9 (18)	
BCOR	Negative	348 (96.1)	323 (96.1)	25 (96.2)	1 f
	Positive	14 (3.9)	13 (3.9)	1 (3.8)	
	missing	341	317	24	
EZH2	Negative	604 (96)	556 (95.7)	48 (100)	0.25 f
	Positive	25 (4)	25 (4.3)	0 (0)	
	missing	74	72	2	
SF3B1	Negative	654 (96)	605 (95.7)	49 (100)	0.25 f
	Positive	27 (4)	27 (4.3)	0 (0)	
	missing	22	21	1	
SRSF2	Negative	608 (89.8)	560 (89.2)	48 (98)	0.049 f
	Positive	69 (10.2)	68 (10.8)	1 (2)	
	missing	26	25	1	
STAG2	Negative	515 (93.3)	481 (93.6)	34 (89.5)	0.31 f
	Positive	37 (6.7)	33 (6.4)	4 (10.5)	
	missing	151	139	12	
U2AF1	Negative	632 (96.9)	586 (96.7)	46 (100)	0.39 f
	Positive	20 (3.1)	20 (3.3)	0 (0)	
	missing	51	47	4	
ZRSR2	Negative	599 (98)	558 (98.2)	41 (95.3)	0.20 f
	Positive	12 (2)	10 (1.8)	2 (4.7)	
	missing	92	85	7	
TP53	Negative	639 (92.5)	591 (91.9)	48 (100)	0.04 f
	Positive	52 (7.5)	52 (8.1)	0 (0)	
	missing	12	10	2	

Abbreviations: wtWT1: wild-type Wilms tumor 1; mWT1: mutated Wilms tumor 1; f- fisher exact test. Results expressed as frequency (%).

Supplemental Table S2: Conditioning regimen details

Variables	Modalities	N=703	wtWT1 (n=653)	mWT1 (n=50)
Conditioning regimen	BuCy based	63 (9)	62 (9.5)	1 (2)
	BuFlu based	221 (31.6)	203 (31.2)	18 (36)
	BuFluThio based	202 (28.9)	191 (29.4)	11 (22)
	FluMel based	88 (12.6)	78 (12)	10 (20)
	TBI based	65 (9.3)	58 (8.9)	7 (14)
	TreoFlu based	54 (7.7)	51 (7.8)	3 (6)
	Other	7 (1)	7 (1.1)	0 (0)
	missing	3	3	0

Abbreviations: wtWT1- wild type Wilms tumor 1; mWT1 – mutated Wilms tumor 1; TBI- total body irradiation; Mel-melphalan; Bu-busulfan; Flu-fludarabine; Thio-thiotepa; Cy- cytoxan; Treo-treosulfan.

Supplemental Table S3: GVHD prophylaxis details

Variables	Modalities	N=703	wtWT1 (n=653)	mWT1 (n=50)
GVHD prevention	CSA based	45 (6.5)	39 (6)	6 (12)
	CSA+MMF based	188 (27)	172 (26.6)	16 (32)
	CSA+MTX	163 (23.4)	154 (23.8)	9 (18)
	CSA+MTX+MMF	14 (2)	14 (2.2)	0 (0)
	MMF+MTX based	22 (3.2)	21 (3.3)	1 (2)
	MMF+SIRO/TACRO based	215 (30.9)	199 (30.8)	16 (32)
	MTX+SIRO/TACRO based	17 (2.4)	16 (2.5)	1 (2)
	SIRO/TACRO based	18 (2.6)	17 (2.6)	1 (2)
	Other	14 (2)	14 (2.2)	0 (0)
	missing	8	8	0

Abbreviations: wtWT1- wild type Wilms tumor 1; mWT1 – mutated Wilms tumor 1; GVHD- graft-versus-host disease; CSA- cyclosporine A; MTX- methotrexate; MMF- mycophenolate mofetil; SIRO- sirolimus; TACRO- tacrolimus.

Supplemental Table S4: Patient and transplant characteristics (matched-pair analysis)

Variables	Modalities	N=176	wtWT1 (n=127)	mWT1 (n=49)
Year of HSCT	median [IQR]<br>(range)	2019 [2018-2021]<br>(2016-2023)	2019 [2018-2021]<br>(2016-2023)	2019 [2018-2020]<br>(2016-2022)
Age at HSCT	median [IQR]<br>(range)	46.22 [35.5-57.2]<br>(19.8-73.2)	46.9 [35.5-57.7]<br>(19.8-73.2)	46 [36.2-56.2]<br>(20.3-70.2)
Patient sex	Female	114 (64.8)	82 (64.6)	32 (65.3)
	Male	62 (35.2)	45 (35.4)	17 (34.7)
Months between diag and HSCT	median [IQR]<br>(range)	4.6 [3.8-5.8]<br>(1.7-23.4)	4.5 [3.9-5.8]<br>(2.1-20.7)	4.7 [3.7-6.2]<br>(1.7-23.4)
AML type	de novo	131 (74.4)	90 (70.9)	41 (83.7)
	secAML	45 (25.6)	37 (29.1)	8 (16.3)
Chromosomal analysis	Abnormal	87 (49.4)	68 (53.5)	19 (38.8)
	Normal	63 (35.8)	42 (33.1)	21 (42.9)
	Not done/Failed	26 (14.8)	17 (13.4)	9 (18.4)
Cytogenetics ELN2022	Favorable	10 (6.9)	7 (6.5)	3 (7.9)
	Intermediate	97 (66.9)	69 (64.5)	28 (73.7)
	Adverse	38 (26.2)	31 (29)	7 (18.4)
	missing	31	20	11
MRD	Positive	37 (33.6)	27 (37)	10 (27)
	Negative	73 (66.4)	46 (63)	27 (73)
	missing	66	54	12
KPS	< 90	30 (17.5)	21 (17.1)	9 (18.8)
	>= 90	141 (82.5)	102 (82.9)	39 (81.2)
	missing	5	4	1
Myeloablative regimen	No	70 (40.2)	49 (39.2)	21 (42.9)
	Yes	104 (59.8)	76 (60.8)	28 (57.1)
	missing	2	2	0
In vivo TCD	No	61 (34.7)	44 (34.6)	17 (34.7)
	ATG	114 (64.8)	82 (64.6)	32 (65.3)
	Campath	1 (0.6)	1 (0.8)	0 (0)
PTCy	No	124 (70.9)	94 (74.6)	30 (61.2)
	Yes	51 (29.1)	32 (25.4)	19 (38.8)
	missing	1	1	0
Source of cells	BM	4 (2.3)	3 (2.4)	1 (2)
	PB	172 (97.7)	124 (97.6)	48 (98)
Donor type	MSD	52 (29.5)	38 (29.9)	14 (28.6)
	Haplo	30 (17)	23 (18.1)	7 (14.3)
	MMR 1 locus	2 (1.1)	0 (0)	2 (4.1)
	UD 10/10	69 (39.2)	51 (40.2)	18 (36.7)
	UD 9/10	23 (13.1)	15 (11.8)	8 (16.3)

Female to Male	No	159 (90.3)	115 (90.6)	44 (89.8)
	Yes	17 (9.7)	12 (9.4)	5 (10.2)
Patient CMV	Negative	49 (28.5)	33 (26.8)	16 (32.7)
	Positive	123 (71.5)	90 (73.2)	33 (67.3)
	missing	4	4	0
Donor CMV	Negative	80 (46)	61 (48.8)	19 (38.8)
	Positive	94 (54)	64 (51.2)	30 (61.2)
	missing	2	2	0

Abbreviations: wtWT1- wild type Wilms tumor 1; mWT1 – mutated Wilms tumor 1; IQR-interquartile range; HSCT-hematopoietic stem cell transplantation; AML-acute myeloid leukemia; secAML-secondary acute myeloid leukemia; diag -diagnosis; CMV-cytomegalovirus; BM-bone marrow; PB-peripheral blood; UD- unrelated donor; MSD-matched sibling donor; Haplo-haploidentical donor; KPS- Karnofsky performance score; TCD- T-cell depletion ; ATG – anti-thymocyte globulin; ELN-European LeukemiaNet; PTCy-post transplantation cyclophosphamide; MRD-measurable residual disease; MMR- mismatch repair.

Unless otherwise specified, results are expressed as n (%)

Supplemental Table S5: Additional mutations (matched-pair analysis)

Variables	Modalities	N=176	wtWT1 (n=127)	mWT1 (n=49)
CEBPA	Negative	124 (91.2)	93 (93.9)	31 (83.8)
	Positive	12 (8.8)	6 (6.1)	6 (16.2)
	missing	40	28	12
FLT3ITD	Negative	131 (76.6)	103 (82.4)	28 (60.9)
	Positive	40 (23.4)	22 (17.6)	18 (39.1)
	missing	5	2	3
NPM1	Negative	143 (84.6)	107 (87)	36 (78.3)
	Positive	26 (15.4)	16 (13)	10 (21.7)
	missing	7	4	3
ASXL1	Negative	167 (94.9)	119 (93.7)	48 (98)
	Positive	9 (5.1)	8 (6.3)	1 (2)
RUNX1	Negative	148 (84.1)	108 (85)	40 (81.6)
	Positive	28 (15.9)	19 (15)	9 (18.4)
BCOR	Negative	97 (97)	72 (97.3)	25 (96.2)
	Positive	3 (3)	2 (2.7)	1 (3.8)
	missing	76	53	23
EZH2	Negative	158 (97.5)	111 (96.5)	47 (100)
	Positive	4 (2.5)	4 (3.5)	0 (0)
	missing	14	12	2
SF3B1	Negative	161 (93.6)	113 (91.1)	48 (100)
	Positive	11 (6.4)	11 (8.9)	0 (0)
	missing	4	3	1

SRSF2	Negative	161 (94.2)	114 (92.7)	47 (97.9)
	Positive	10 (5.8)	9 (7.3)	1 (2.1)
	missing	5	4	1
STAG2	Negative	128 (94.1)	95 (96)	33 (89.2)
	Positive	8 (5.9)	4 (4)	4 (10.8)
	missing	40	28	12
U2AF1	Negative	163 (99.4)	118 (99.2)	45 (100)
	Positive	1 (0.6)	1 (0.8)	0 (0)
	missing	12	8	4
ZRSR2	Negative	147 (97.4)	107 (98.2)	40 (95.2)
	Positive	4 (2.6)	2 (1.8)	2 (4.8)
	missing	25	18	7
TP53	Negative	163 (94.8)	116 (92.8)	47 (100)
	Positive	9 (5.2)	9 (7.2)	0 (0)
	missing	4	2	2

Abbreviations: wtWT1- wild type Wilms tumor 1; mWT1 – mutated Wilms tumor 1.

Results are expressed as n (%).

Supplemental Table S6: Conditioning regimen details (matched-pair analysis)

Variables	Modalities	N=176	wtWT1 (n=127)	mWT1 (n=49)
Conditioning regimen	BuCy based	18 (10.3)	18 (14.3)	0 (0)
	BuFlu based	58 (33.1)	40 (31.7)	18 (36.7)
	BuFluThio based	43 (24.6)	32 (25.4)	11 (22.4)
	FluMel based	22 (12.6)	12 (9.5)	10 (20.4)
	TBI based	19 (10.9)	12 (9.5)	7 (14.3)
	TreoFlu based	13 (7.4)	10 (7.9)	3 (6.1)
	Other	2 (1.1)	2 (1.6)	0 (0)
	missing	1	1	0

Abbreviations: wtWT1- wild type Wilms tumor 1; mWT1 – mutated Wilms tumor 1; TBI-total body irradiation; Mel-melphalan; Bu-busulfan; Flu-fludarabine; Cy-cytoxan; Treo-treosulfan. Results are expressed as n (%).

Supplemental Table S7: GVHD prevention (matched-pair analysis)

Variables	Modalities	N=176	wtWT1 (n=127)	mWT1 (n=49)
GVHD prevention	CSA based	19 (10.8)	13 (10.2)	6 (12.2)
	CSA+MMF based	51 (29)	35 (27.6)	16 (32.7)
	CSA+MTX	51 (29)	43 (33.9)	8 (16.3)
	CSA+MTX+MMF	2 (1.1)	2 (1.6)	0 (0)
	MMF+MTX based	3 (1.7)	2 (1.6)	1 (2)
	MMF+SIRO/TACRO based	39 (22.2)	23 (18.1)	16 (32.7)
	MTX+SIRO/TACRO based	4 (2.3)	3 (2.4)	1 (2)
	SIRO/TACRO based	4 (2.3)	3 (2.4)	1 (2)
	Other	3 (1.7)	3 (2.4)	0 (0)

Abbreviations: wtWT1- wild type Wilms tumor 1; mWT1 – mutated Wilms tumor 1; CSA- cyclosporine A; MTX- methotrexate; MMF- mycophenolate mofetil; SIRO- sirolimus; TACRO- tacrolimus. Results are expressed as n (%).
